# Immune responses of different COVID-19 vaccination strategies by analyzing single-cell RNA sequencing data from multiple tissues using machine learning methods

**DOI:** 10.3389/fgene.2023.1157305

**Published:** 2023-03-17

**Authors:** Hao Li, Qinglan Ma, Jingxin Ren, Wei Guo, Kaiyan Feng, Zhandong Li, Tao Huang, Yu-Dong Cai

**Affiliations:** ^1^ College of Food Engineering, Jilin Engineering Normal University, Changchun, China; ^2^ School of Life Sciences, Shanghai University, Shanghai, China; ^3^ Key Laboratory of Stem Cell Biology, Shanghai Institutes for Biological Sciences (SIBS), Shanghai Jiao Tong University School of Medicine (SJTUSM), Chinese Academy of Sciences (CAS), Shanghai, China; ^4^ Department of Computer Science, Guangdong AIB Polytechnic College, Guangzhou, China; ^5^ Bio-Med Big Data Center, CAS Key Laboratory of Computational Biology, Shanghai Institute of Nutrition and Health, University of Chinese Academy of Sciences, Chinese Academy of Sciences, Shanghai, China; ^6^ CAS Key Laboratory of Tissue Microenvironment and Tumor, Shanghai Institute of Nutrition and Health, University of Chinese Academy of Sciences, Chinese Academy of Sciences, Shanghai, China

**Keywords:** immune response, COVID-19 vaccination, SARS-CoV-2 infection, machine learning method, classification rule

## Abstract

Multiple types of COVID-19 vaccines have been shown to be highly effective in preventing SARS-CoV-2 infection and in reducing post-infection symptoms. Almost all of these vaccines induce systemic immune responses, but differences in immune responses induced by different vaccination regimens are evident. This study aimed to reveal the differences in immune gene expression levels of different target cells under different vaccine strategies after SARS-CoV-2 infection in hamsters. A machine learning based process was designed to analyze single-cell transcriptomic data of different cell types from the blood, lung, and nasal mucosa of hamsters infected with SARS-CoV-2, including B and T cells from the blood and nasal cavity, macrophages from the lung and nasal cavity, alveolar epithelial and lung endothelial cells. The cohort was divided into five groups: non-vaccinated (control), 2*adenovirus (two doses of adenovirus vaccine), 2*attenuated (two doses of attenuated virus vaccine), 2*mRNA (two doses of mRNA vaccine), and mRNA/attenuated (primed by mRNA vaccine, boosted by attenuated vaccine). All genes were ranked using five signature ranking methods (LASSO, LightGBM, Monte Carlo feature selection, mRMR, and permutation feature importance). Some key genes that contributed to the analysis of immune changes, such as *RPS23*, *DDX5*, *PFN1* in immune cells, and *IRF9* and *MX1* in tissue cells, were screened. Afterward, the five feature sorting lists were fed into the feature incremental selection framework, which contained two classification algorithms (decision tree [DT] and random forest [RF]), to construct optimal classifiers and generate quantitative rules. Results showed that random forest classifiers could provide relative higher performance than decision tree classifiers, whereas the DT classifiers provided quantitative rules that indicated special gene expression levels under different vaccine strategies. These findings may help us to develop better protective vaccination programs and new vaccines.

## 1 Introduction

Since the outbreak of a novel coronavirus, known as Severe Acute Respiratory Syndrome Coronavirus 2 (SARS-CoV-2), in late 2019, there has been an unprecedented global impact. In particular, as of 28 September 2022, more than 616 million cases have been diagnosed, and more than 6.5 million deaths have been reported worldwide (Fabbri et al., 2014). The World Health Organization named the disease caused by SARS-CoV-2 as coronavirus disease 2019 (COVID-19). Fever, sore throat, dry cough, and symptoms of pneumonia are common clinical manifestations of COVID-19, and severe COVID-19 can even lead to death (Guan et al., 2020; Parasher, 2021). Licensed vaccines have proven highly effective in preventing symptomatic and asymptomatic SARS-CoV-2 infections and reducing COVID-19-related hospitalizations and deaths (Haas et al., 2021; Castro Dopico et al., 2022), and they have given the world hope to defeat SARS-CoV-2.

A variety of COVID-19 vaccines have been marketed in response to the massive spread of SARS-CoV-2, such as mRNA vaccines, inactivated/attenuated whole virus vaccines, adenovirus vector vaccines, and recombinant protein vaccines. mRNA vaccines such as the widely used BNT16b2, which contains mRNA that can encode the SARS-CoV-2 spike protein (Mabrouk et al., 2022), have been reported effective against infection, with effectivity accounting for 89.5%–99.2% against alpha variants, 75%–96.4% against beta, and 42%–84.4% against delta (Fiolet et al., 2022). Attenuated vaccines have been used against measles virus, rubella virus, and influenza virus (Okamura and Ebina, 2021). Viruses with slow rates of proliferation in the human body were mostly attenuated through adaptation to cold culture conditions. (Makino et al., 1974; Parks et al., 2001). Live-attenuated vaccines can induce immune responses against multiple antigens and activate higher mucosal immune responses compared with other current COVID-19 vaccines (Han et al., 2021; Okamura and Ebina, 2021), which have a better and long-lasting immune effect. In general, adenoviral vector vaccines modify replication-deficient adenoviruses to express SARS-CoV-2 S protein or its epitopes (Feng et al., 2020). Viral vector vaccines can combine the safety benefits of inactivated vaccines with the immunological benefits of attenuated vaccines (Baron et al., 2018). For example, ChAdOx1 has been reported to have 74.5% protection against alpha and 67.0% protection against delta (Lopez Bernal et al., 2021).

Almost all approved COVID-19 vaccines are effective in inducing protective systemic immunity, including the induction of T-cell responses (cellular immunity) (Oberhardt et al., 2021; Wherry and Barouch, 2022) and B-cell responses (antibody immunity) (Turner et al., 2021), along with the production of long-lived memory T cells and memory B cells (Sette and Crotty, 2022). Vaccine composition and dose can potentially affect the development of different immune responses. “Homologous prime-boost” vaccination is when subjects are given the same type of vaccine in a second dose as the first (Mahase, 2021), whereas “heterologous prime-boost” vaccination is when different vaccine strategies are combined in the primary and booster phases (He et al., 2021). The majority of studies have concluded that “heterologous prime-boost” vaccination has a protective immunological advantage over “homologous prime-boost” vaccination (Benning et al., 2021; Fabricius et al., 2021; Gao et al., 2022), whereas “heterologous prime-boost” immunization may induce severe side effects (Liu X. et al., 2021; Hillus et al., 2021). However, few writers have been able to draw on any systematic comparison of “homologous prime-boost” vaccination and “heterologous prime-boost” vaccination.

This study was designed to compare the protective capacity of different vaccination strategies, including mRNA vaccine, adenoviral vector vaccine, and modified live-attenuated vaccine. The mRNA vaccine BNT16b2 and the adenovirus vaccine ChAdOx1 have received the majority of attention in recent studies, whereas comparison studies on attenuated vaccine are limited. Cell samples in eight cell types from the blood, lungs, and nasal mucosa of Syrian hamsters were divided into five groups: non-vaccinated (control), 2*adenovirus (two doses of adenovirus vaccine), 2*attenuated (two doses of attenuated virus vaccine), 2*mRNA (two doses of mRNA vaccine), and mRNA/attenuated (primed by mRNA vaccine, boosted by attenuated vaccine). Based on single-cell data on gene expression in Syrian hamsters infected with SARS-CoV-2 by nasal drip 21 days after two doses of vaccine, machine learning based analysis was designed to explore differences in immune memory protection induced by different prime-boost vaccination strategies and target cell immune status after SARS-CoV-2 infection. Five feature ranking algorithms: least absolute shrinkage and selection operator (LASSO) (Ranstam and Cook, 2018), light gradient boosting machine (LightGBM) (Ke et al., 2017), Monte Carlo feature selection (MCFS) (Dramiński and Koronacki, 2018), max-relevance and min-redundancy (mRMR) (Peng et al., 2005), and permutation feature importance (PFI) (Fisher et al., 2019) were applied to the single-cell data on each cell type, yielding five feature lists. These lists were fed into incremental feature selection (IFS) (Liu and Setiono, 1998), which incorporated decision tree (DT) (Safavian and Landgrebe, 1991) and random forest (RF) (Breiman, 2001), to extract important features, build effective classifiers and classification rules. The classifier and rules can be used to monitor the level of immunity and disease risk in SARS-CoV-2-infected patients following different vaccine combination. The features (e.g., *RPS23*, *DDX5*, *PFN1* in immune cells, and *IRF9* and *MX1* in tissue cells) and rules identified in this study could be helpful in the research for prime-boost vaccination methods, providing improved protection and duration.

## 2 Materials and methods

**FIGURE 1:**
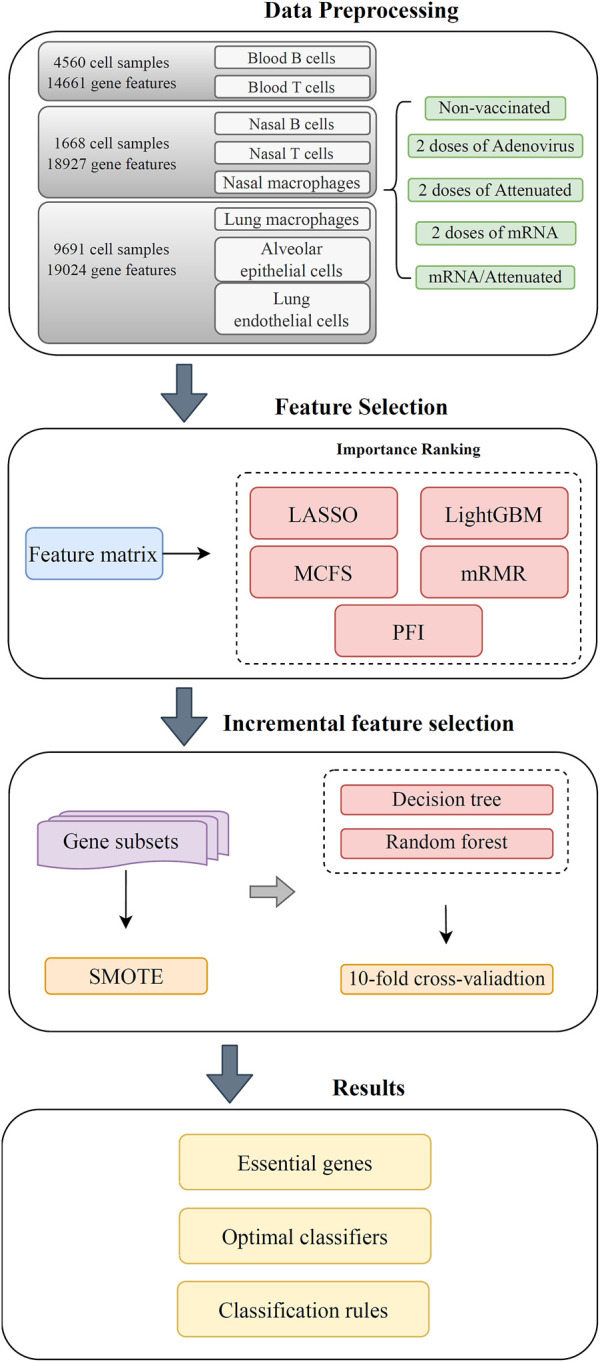
Flow chart of the entire computational analysis. Gene expression profiling data of SARS-CoV-2 infection in hamster were analyzed using a machine learning based approach with samples from blood T cells, blood B cells, nasal T cells, nasal B cells, lung macrophages, nasal macrophages, alveolar epithelial cells, and lung endothelial cells. Each cell has five vaccination states, that is, unvaccinated, two doses of adenovirus vaccine, two doses of attenuated virus vaccine, two doses of mRNA vaccine, and one dose of mRNA followed by one dose of attenuated vaccine. Gene features were analyzed by five feature selection methods, namely, LASSO, LightGBM, MCFS, mRMR, and PFI. The resulting feature lists were fed into the incremental feature selection (IFS) method to extract the underlying genes, construct effective classifiers and classification rules.

The entire workflow used in this study is shown in Figure 1. After grouping the obtained expression profile data on each cell type, the genes were ranked using several feature ranking algorithms, and a number of ranked lists were generated. Then, each list was fed into the IFS method with DT or RF. Two optimal classifiers were constructed. The methods involved are described in detail in this section.

### 2.1 Data

Expression profiling data for different cell types from Syrian hamsters were obtained from the GEO database under accession number GSE200596 (Nouailles et al., 2022). These data describe the cellular response to SARS-CoV-2 infection in hamsters vaccinated with mRNA vaccine, adenovirus vaccine, and attenuated virus vaccine for 21 days. Data were obtained from immune cells and tissue cells, including blood T cells, blood B cells, nasal T cells, nasal B cells, lung macrophages, nasal macrophages, alveolar epithelial and lung endothelial cells. Cell samples in each cell type were divided into five groups based on vaccination status: non-vaccine group (control group), 2*adenovirus group (two doses of adenovirus vaccine), 2*attenuated group (two doses of attenuated virus vaccine sCPD9), 2*mRNA group (two doses of mRNA vaccine), and mRNA/attenuated group (primed by mRNA vaccine and boosted by attenuated vaccine sCPD9). Table 1 demonstrates the number of cells in each group for eight cell types. Each sample from the blood, nasal cavity, and lungs contained 14661, 18927, and 19024 genes, respectively. Using genes as features and five groups as sample labels, they were entered into a machine learning framework for the analysis of the classification problem.

**TABLE 1:** Sample size of five vaccination strategies from eight cell types.

Cell type	COVID-19 vaccination strategy	Sample size
Blood B cells	Non-vaccinated	316
	Adenovirus vaccine + Adenovirus vaccine	524
	Attenuated vaccine + Attenuated vaccine	1,018
	mRNA vaccine + mRNA vaccine	547
	mRNA vaccine + Attenuated vaccine	722
Blood T cells	Non-vaccinated	90
	Adenovirus vaccine + Adenovirus vaccine	242
	Attenuated vaccine + Attenuated vaccine	523
	mRNA vaccine + mRNA vaccine	272
	mRNA vaccine + Attenuated vaccine	306
Nasal B cells	Non-vaccinated	88
	Adenovirus vaccine + Adenovirus vaccine	38
	Attenuated vaccine + Attenuated vaccine	20
	mRNA vaccine + mRNA vaccine	61
	mRNA vaccine + Attenuated vaccine	96
Nasal T cells	Non-vaccinated	54
	Adenovirus vaccine + Adenovirus vaccine	43
	Attenuated vaccine + Attenuated vaccine	12
	mRNA vaccine + mRNA vaccine	45
	mRNA vaccine + Attenuated vaccine	61
Nasal macrophages	Non-vaccinated	417
	Adenovirus vaccine + Adenovirus vaccine	303
	Attenuated vaccine + Attenuated vaccine	45
	mRNA vaccine + mRNA vaccine	180
	mRNA vaccine + Attenuated vaccine	205
Lung macrophages	Non-vaccinated	1,437
	Adenovirus vaccine + Adenovirus vaccine	804
	Attenuated vaccine + Attenuated vaccine	753
	mRNA vaccine + mRNA vaccine	1,030
	mRNA vaccine + Attenuated vaccine	645
Alveolar epithelial cells	Non-vaccinated	614
	Adenovirus vaccine + Adenovirus vaccine	481
	Attenuated vaccine + Attenuated vaccine	481
	mRNA vaccine + mRNA vaccine	356
	mRNA vaccine + Attenuated vaccine	286
Lung endothelial cells	Non-vaccinated	869
	Adenovirus vaccine + Adenovirus vaccine	527
	Attenuated vaccine + Attenuated vaccine	724
	mRNA vaccine + mRNA vaccine	362
	mRNA vaccine + Attenuated vaccine	322

### 2.2 Feature ranking algorithms

Each sample was represented by a large number of features. It is necessary to understand which of these genes are associated with COVID-19 vaccination and SARS-CoV-2 infection. The genes involved in each cell type were analyzed using five ranking algorithms and sorted by their importance. These algorithms included LASSO (Ranstam and Cook, 2018), LightGBM (Ke et al., 2017), MCFS (Dramiński and Koronacki, 2018), mRMR (Peng et al., 2005), and PFI (Fisher et al., 2019). These methods have been widely practiced in solving life science problems (Zhao et al., 2018; Li et al., 2022a; Li et al., 2022b; Li Z. et al., 2022; Lu et al., 2022; Huang et al., 2023a; Huang et al., 2023b).

#### 2.2.1 Least absolute shrinkage and selection operator

LASSO is a regression analysis method that can accomplish feature selection. It inputs the feature matrix into a first-order penalty function that treats the features as independent variables. This penalty function contains L1-type regularization terms. After optimization, features that tend to contribute more greatly affect the outcome of the function, a process is executed to adjust the coefficients of the independent variable. Consequently, the coefficients of some features decrease to zero, which are considered as redundant features by the algorithm and eliminated. The magnitude of the absolute value of the coefficients of the independent variables is picked up to determine the importance of the corresponding features. Accordingly, features can be ranked in a list. To execute LASSO, the package collected in Scikit-learn (Pedregosa et al., 2011) was used in this study. Default parameters were adopted.

#### 2.2.2 Light gradient boosting machine

The LightGBM method is derived from the gradient boosting DT, which is a tree structure. It is suitable for handling high-dimensional data because it can bundle mutually exclusive features during computation. A leaf-wise growth strategy was used to determine the attributes of the instances, and only the branches with high efficiency were extended. Therefore, the higher the degree of participation in the construction of the tree, the higher the degree of feature contribution it represents. Thus, features can be ranked in accordance with the degree of involvement. The present study adopted the LightGBM program obtained from https://lightgbm.readthedocs.io/en/latest/. For convenience, it was executed with default parameters.

#### 2.2.3 Monte Carlo feature selection

The MCFS method is executed by constructing a number of independent DTs. The features and training samples used to build these trees are randomly selected. The random selection yields p subsets of features, and for each feature subset, *t* datasets are built by randomly splitting training and test samples. A DT is built on each dataset. Thus, p×t classification trees can be constructed. The importance of each feature is expressed using the relative importance (RI) score, which can be computed by

$$RI_{g}=\overset{p\times t}{\underset{\tau =1}{\sum }}{\left(\omega A_{CC}\right)}^{u}\underset{ng\left(\tau \right)}{\sum }IG\left(ng\left(\tau \right)\right){\left(\frac{no.in\;ng\left(\tau \right)}{no.in\;\tau }\right)}^{v},$$ (1)

In the formula, $\omega A_{CC}$ is the weighted accuracy of the tree τ ; $ng\left(\tau \right)$ is a node in the tree τ whose information gain is denoted as $IG\left(ng\left(\tau \right)\right)$ , and $no.in\;ng\left(\tau \right)$ denotes the sample size of $ng\left(\tau \right)$ , no.in τ denotes the sample size in the root of τ . In addition, u and v are two positive numbers weighting $\omega A_{CC}$ and $no.in\;ng\left(\tau \right)/no.in\;\tau$ , respectively. The higher the RI score of a feature, the more important it is. Features can be sorted in a list with the decreasing order of their RI values. The MCFS program was retrieved from http://www.ipipan.eu/staff/m.draminski/mcfs.html, which was performed with default parameters.

#### 2.2.4 Max-relevance and min-redundancy

mRMR aims to select features that are least correlated with other features but have maximum correlation with the target variable. The correlation between the features and target variable and the redundancy between features are all measured by mutual information (MI). It first creates an empty list of features and selects one feature in each round. Generally, the feature with the highest correlation to target variable and lowest redundancy to features already in the list is selected and appended to the list. The process is repeated until all features are in the list. The mRMR package adopted in this study was obtained from http://home.penglab.com/proj/mRMR/. It was run using default parameters.

#### 2.2.5 Permutation feature importance

RF is a powerful classification algorithm. It can also be used to evaluate the importance of features. Its logic is simple. If the values of a feature are permutated randomly in such a way that it causes a larger prediction error, then the feature is more important. Conversely, if it does not cause a change in the prediction result, then the feature is considered unimportant. Features are ranked in a list in terms of the change of prediction error. Here, the PFI program was downloaded from scikit-learn (Pedregosa et al., 2011). It was performed with default parameters.

Above feature ranking algorithms were applied to the expression profiling data on each cell type. For easy descriptions, the lists generated by these five algorithms were called LASSO, LightGBM, MCFS, mRMR and PFI feature lists.

### 2.3 Incremental feature selection

Above five algorithms only sorted features in five lists, which did not tell us which features can be picked up for setting up classifiers. However, these lists had a common trait, that is, features with high ranks were more important than others. This indicated that some top features in the list can be used to build a classifier with good performance. In view of this, the IFS method (Liu and Setiono, 1998) was employed in this study, which can determine the features that achieve the best classification performance for one classification algorithm. It transforms the feature list into a series of feature subsets, where the features in each subset are taken from the top ones of the list, but each subset contains a different number of features. The number of features in each subset is incremented by a step compared with the previous subset. For example, if the step is 10, the first subset contains the first 10 features of the list, the second subset contains the first 20 features of the list, and so on. Then, these subsets are fed into one classification algorithm to construct classifiers, and their performance is evaluated using 10-fold cross-validation (Kohavi, 1995). The performance of these classifiers is observed, and the optimal classifier is selected, at which point the feature subset is the optimal feature subset.

### 2.4 Synthetic minority oversampling technique

The sample sizes were not consistent across inoculation strategies, for example, in the nasal macrophage dataset, the sample size of the non-vaccine group was 9.3 times larger than that of the two* attenuated group. These unbalanced data sets lead to preferences in the results of the classifier. The synthetic minority oversampling technique (SMOTE) method (Chawla et al., 2002) was used to tackle such problem in this study. It adds new samples to minority classes for enlarging its size. In detail, SMOTE randomly selects a sample from a minority class and then determines the k nearest samples of the selected sample in the same class using the Euclidean distance as a metric. On the line segment between one randomly selected nearest neighbor and the current sample, a random point is selected and treated as a newly generated sample. This process is repeated until the data set is balanced. Here, we used the program downloaded from https://github.com/scikit-learn-contrib/imbalanced-learn to implement SMOTE. The default parameters were adopted.

### 2.5 Classification algorithm

As previously described, IFS must be coupled with a classification algorithm. In this study, DT (Safavian and Landgrebe, 1991; Zhang et al., 2021a; Zhang et al., 2021b) and RF (Breiman, 2001; Chen et al., 2021; Ran et al., 2022; Yang and Chen, 2022; Wang and Chen 2023) were used to construct the classifiers. Their brief introduction is as below.

#### 2.5.1 Random forest

RF is one of the most classic classification algorithms in machine learning. In fact, it is an ensemble algorithm, which contains several DTs. Each tree is constructed by randomly selecting samples and features and the selected samples are as many as the training samples but can be same for some samples. For a test sample, each tree provides its decision. The result of RF is determined in accordance with the majority rule on all decisions. To implement RF, the corresponding package in scikit-learn (Pedregosa et al., 2011) was employed. For convenience, it was performed with default parameters.

#### 2.5.2 Decision tree

Although RF is a powerful classification algorithm, the underlying classification principle is difficult to capture as it is a black-box algorithm. In this case, few medical insights can be obtained. DT is a classic white-box algorithm as the classification procedures are completely open, which provides more opportunities to understand the classification principle. It can be represented by a tree, where each internal node represents a feature with a threshold and each leaf node indicates the predicted result (class label). In addition to the tree representation, DT can also be represented by a group of rules. Each rule is generated by a path from the root to one leaf node. These rules imply the essential clues hidden in the investigated dataset. Similar to RF, the DT package in scikit-learn (Pedregosa et al., 2011) was employed to construct DT classifiers in IFS method.

### 2.6 Performance evaluation

The F1-measure is often used in machine learning to evaluate the performance of classifiers (Powers, 2011; Liang et al., 2020; Tang and Chen, 2022; Wu and Chen, 2022; Li et al., 2023; Wu and Chen, 2023). For multi-classification problems, F1-measure is defined for each class, which can be computed by

$${Precision}_{i}=\frac{{TP}_{i}}{{TP}_{i}+{FP}_{i}}$$ (2)

$${Recall}_{i}=\frac{{TP}_{i}}{{TP}_{i}+{FN}_{i}}$$ (3)

$${F1-measure}_{i}=\frac{{2\times Precision}_{i}\times {Recall}_{i}}{{Precision}_{i}+{Recall}_{i}}$$ (4)

where ${TP}_{i}$ , ${FP}_{i}$ and ${FN}_{i}$ represent true positive, false positive, and false negative for the *i*th class, *i* is the index of one class. To evaluate the overall performance of the classifiers, the F1-measure values on all classes can be integrated, inducing two measurements: macro F1 and weighted F1. Macro F1 is the direct average of all F1-measure values, whereas weighted F1 further considered the weights of F1-measure values on different classes. The weighted F1 can be expressed by

$$Weighted\;F1=\overset{L}{\underset{i}{\sum }}w_{i}\times {F1-measure}_{i}$$ (5)

where L represents the number of classes and $w_{i}$ represents the proportion of samples in the *i*th class to overall sample.

In addition, prediction accuracy (ACC) and Matthews correlation coefficients (Matthews, 1975; Gorodkin, 2004; Wang and Chen, 2022) were also used for evaluation. ACC is one of the most widely used measurements, which is defined as the proportion of correctly predicted samples. However, such measurement is not very accurate when the dataset is imbalanced. For such dataset, MCC is a more objective measurement. It can be computed by

$$MCC=\frac{cov\left(X,Y\right)}{\sqrt{cov\left(X,X\right)cov\left(Y,Y\right)}}$$ (6)

where *X* and *Y* are two matrices, indicating the true and predicted classes of all samples, $cov\left(X,Y\right)$ stands for the correlation coefficient of two matrices.

## 3 Results

### 3.1 Feature ranking results

The expression profiling data on each cell type was analyzed by five feature ranking algorithms. Each algorithm yielded one feature list. Totally, five feature lists (LASSO, LightGBM, MCFS, mRMR and PFI feature lists) were obtained for each cell type. All these lists on eight cell types are provided in Supplementary Table S1.

### 3.2 Results of incremental feature selection

**FIGURE 2:**
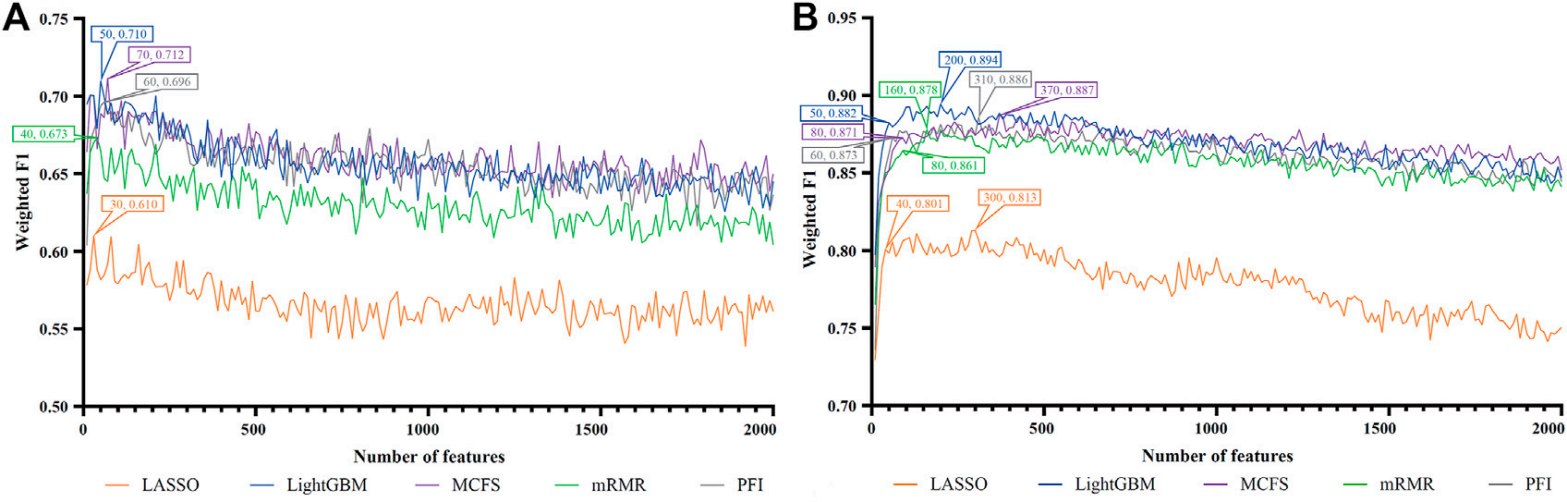
IFS curves of two classification algorithms on five feature lists for blood B cells. **(A)** IFS curves of the decision tree (DT). **(B)** IFS curves of the random forest (RF). The best DT/RF classifier used top 70/200 features in the MCFS/LightGBM feature list.

**FIGURE 3:**
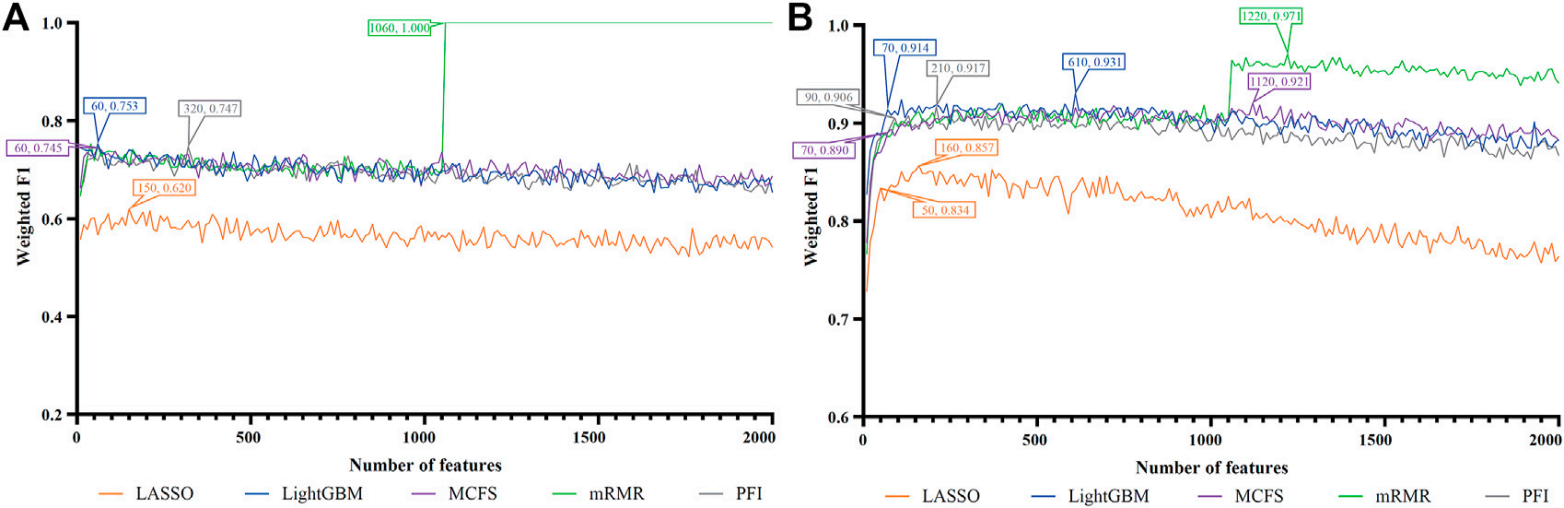
IFS curves of two classification algorithms on five feature lists for blood T cells. **(A)** IFS curves of the decision tree (DT). **(B)** IFS curves of the random forest (RF). The best DT/RF classifier used top 1,060/1,220 features in the mRMR/mRMR feature list.

**FIGURE 4:**
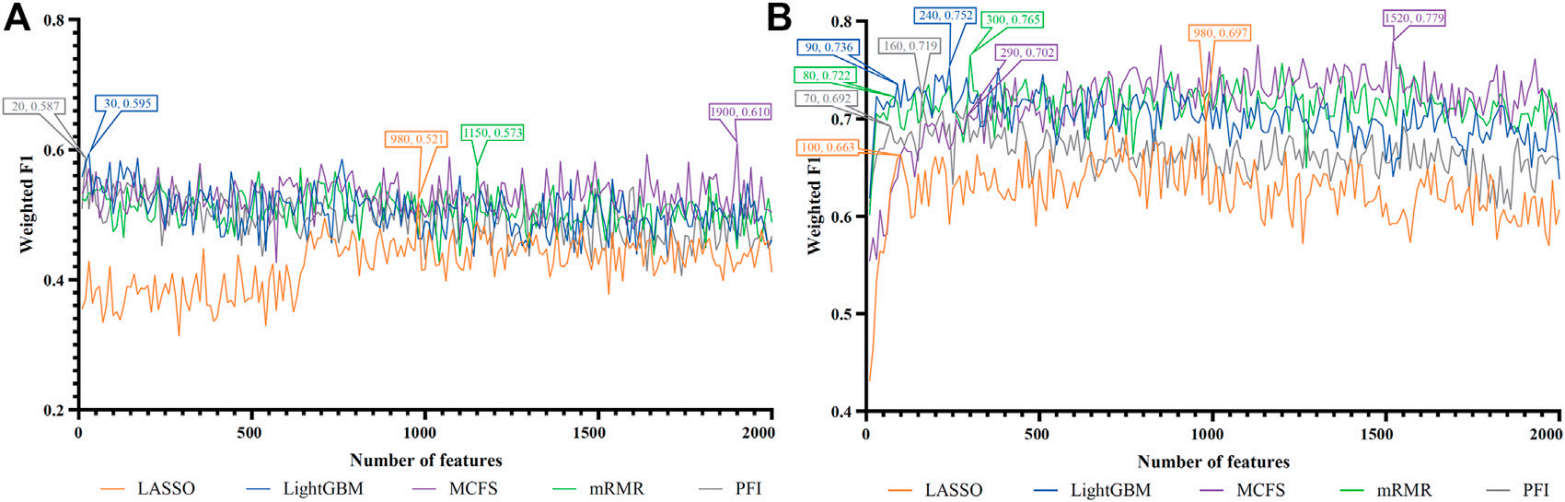
IFS curves of two classification algorithms on five feature lists for nasal B cells. **(A)** IFS curves of the decision tree (DT). **(B)** IFS curves of the random forest (RF). The best DT/RF classifier used top 1900/1,520 features in the MCFS/MCFS feature list.

**FIGURE 5:**
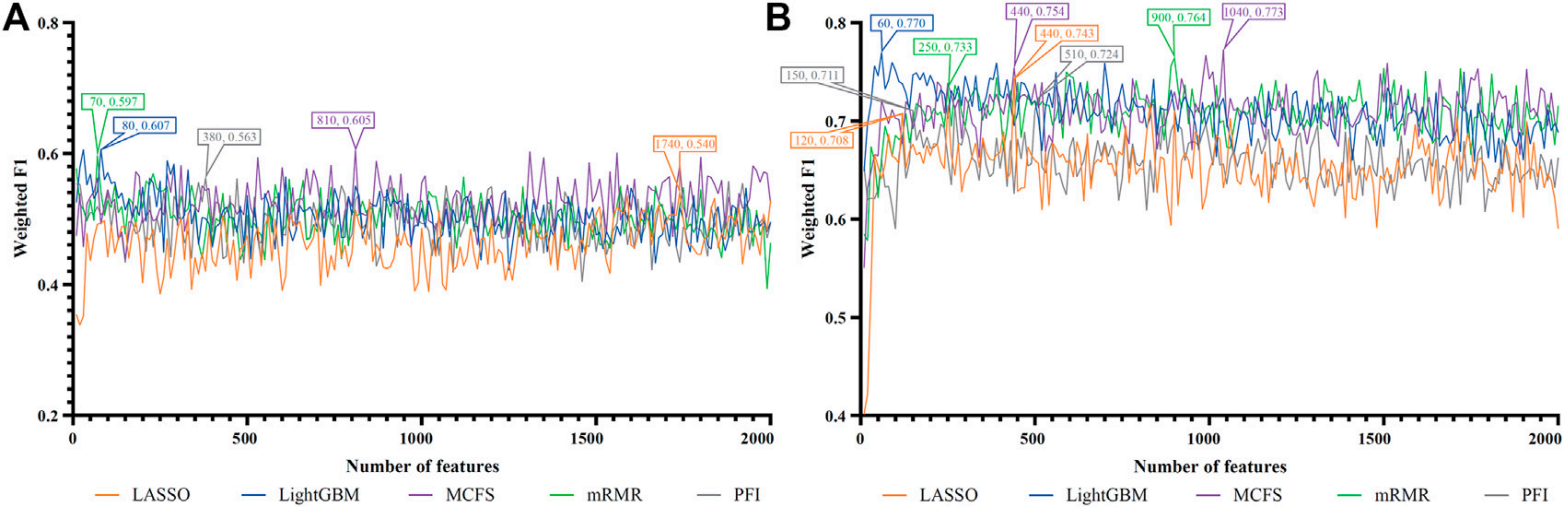
IFS curves of two classification algorithms on five feature lists for nasal T cells. **(A)** IFS curves of the decision tree (DT). **(B)** IFS curves of the random forest (RF). The best DT/RF classifier used top 80/1,040 features in the LightGBM/MCFS feature list.

**FIGURE 6:**
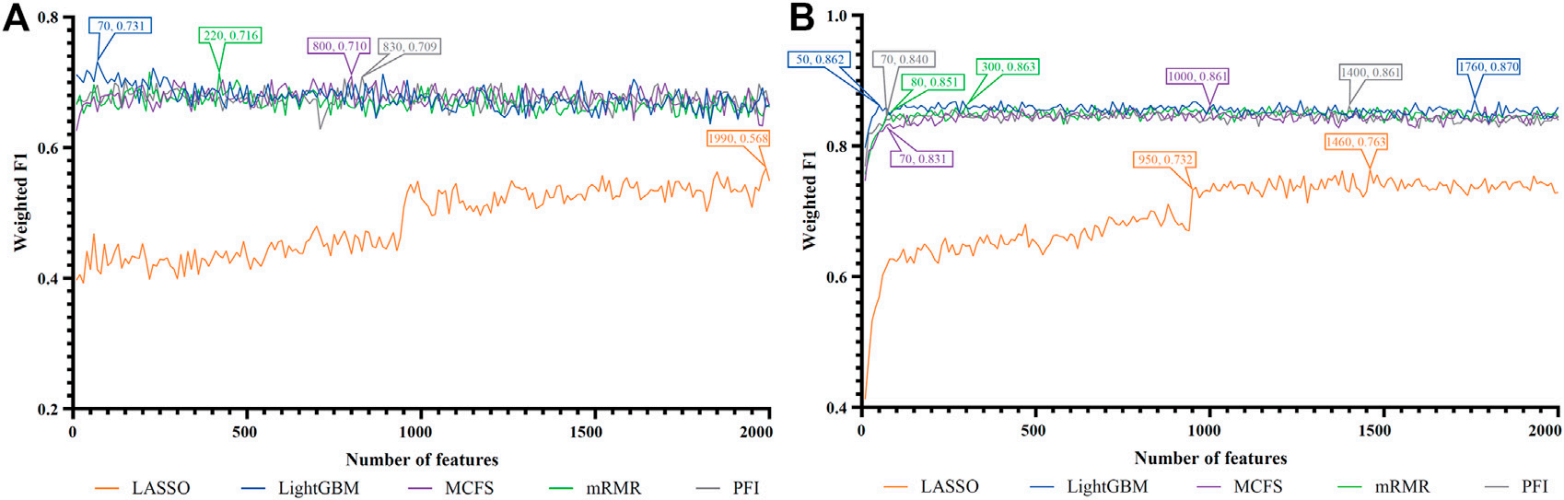
IFS curves of two classification algorithms on five feature lists for nasal macrophages. **(A)** IFS curves of the decision tree (DT). **(B)** IFS curves of the random forest (RF). The best DT/RF classifier used top 70/1760 features in the LightGBM/LightGBM feature list.

**FIGURE 7:**
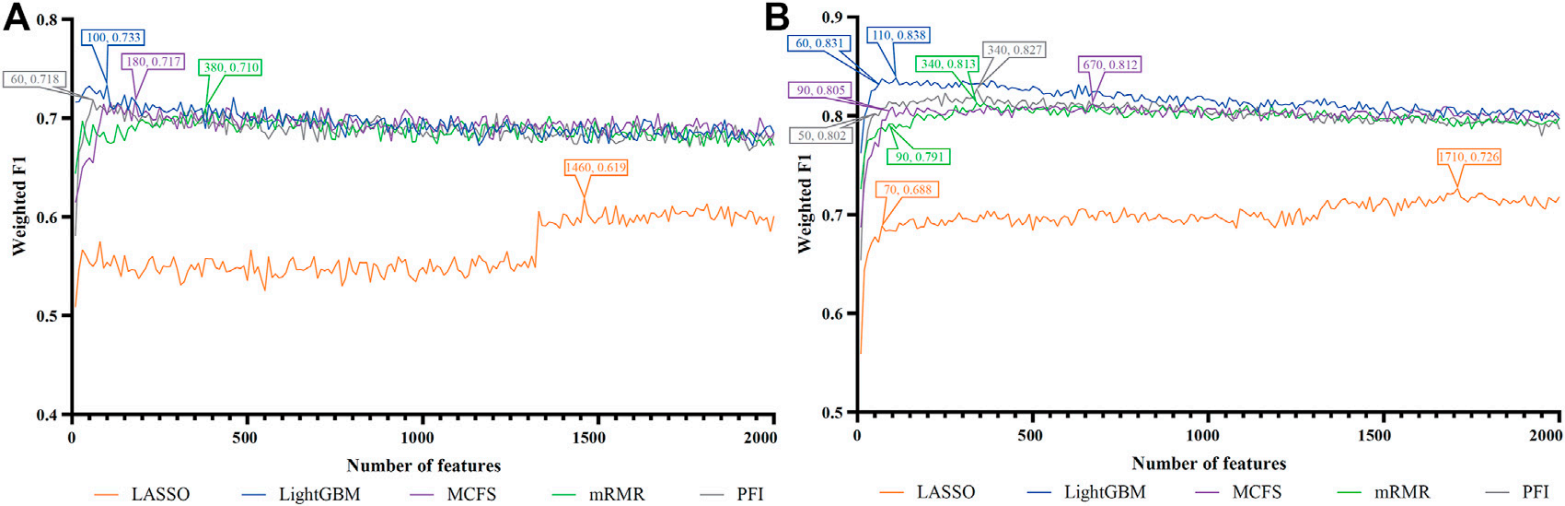
IFS curves of two classification algorithms on five feature lists for lung macrophages. **(A)** IFS curves of the decision tree (DT). **(B)** IFS curves of the random forest (RF). The best DT/RF classifier used top 100/110 features in the LightGBM/LightGBM feature list.

**FIGURE 8:**
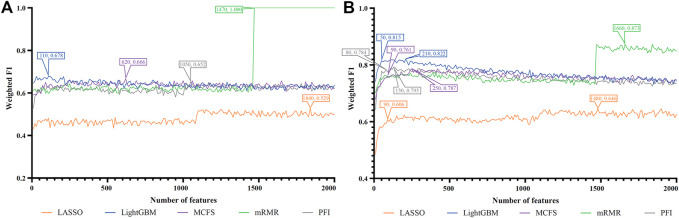
IFS curves of two classification algorithms on five feature lists for lung alveolar epithelial cells. **(A)** IFS curves of the decision tree (DT). **(B)** IFS curves of the random forest (RF). The best DT/RF classifier used top 1,470/1,660 features in the mRMR/mRMR feature list.

**FIGURE 9:**
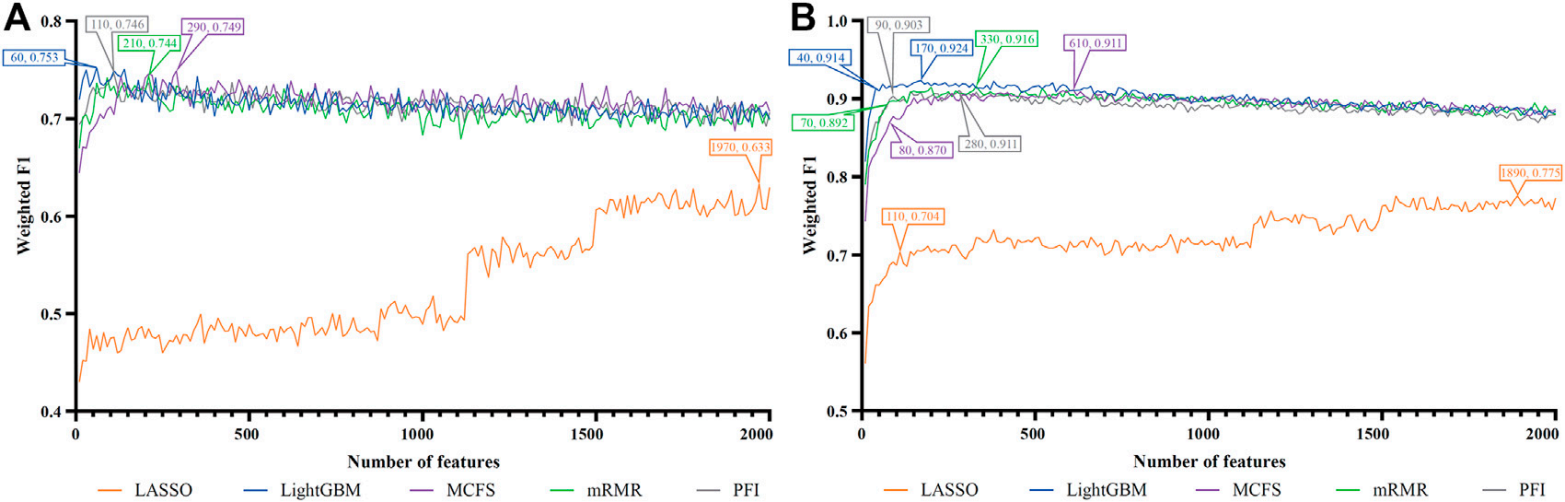
IFS curves of two classification algorithms on five feature lists for lung endothelial cells. **(A)** IFS curves of the decision tree (DT). **(B)** IFS curves of the random forest (RF). The best DT/RF classifier used top 60/170 features in the LightGBM/LightGBM feature list.

For each cell type, five feature lists were obtained, as listed in Supplementary Table S1. Each list was fed into IFS workflow one by one. Although huge number of features were included in each list, only a few features may be highly related to indicate the differences on immune responses of different vaccination status. Thus, it was not necessary to consider all features in the list. Here, we focused on the top 2000 features in each list and adopted step 10 to construct feature subsets in IFS method. Accordingly, 200 feature subsets were constructed, on each of which one DT classifier and one RF classifier were set up. SMOTE was employed to tackle imbalanced problem when building each classifier. All classifiers were evaluated by 10-fold cross-validation. Detailed evaluation results are shown in the Supplementary Table S2. Weighted F1 was selected as the major measurement. Several IFS curves were plotted to show the performance of DT and RF under different numbers of top features in each list, as shown in Figures 2–9.

#### 3.2.1 IFS results of immune cells

For blood B cells, the IFS curves of DT and RF are illustrated in Figures 2A, B, respectively. It can be observed from Figure 2A that DT classifier with the top 70 features in the MCFS feature list can generate the highest weighted F1 of 0.712. As for RF, the best RF classifier adopted the top 200 features in the LightGBM feature list (Figure 2B). The detailed performance of above two classifiers is listed in Table 2. Clearly, the best RF classifier was superior to the best DT classifier. Furthermore, IFS results with RF were generally better than those with DT.

**TABLE 2:** Performance of the best classifiers for eight cell types based on two classification algorithms.

Cell type	Classification algorithm (Feature list)	Number of features	Weight F1	MCC	ACC
Blood B cells	DT (MCFS feature list)	70	0.712	0.627	0.71
	RF (LightGBM feature list)	200	0.894	0.863	0.894
Blood T cells	DT (mRMR feature list)	1,060	1.000	1.000	1.000
	RF (mRMR feature list)	1,220	0.971	0.961	0.971
Nasal B cells	DT (MCFS feature list)	1900	0.610	0.483	0.611
	RF (MCFS feature list)	1,520	0.779	0.706	0.779
Nasal T cells	DT (LightGBM feature list)	80	0.607	0.489	0.605
	RF (MCFS feature list)	1,040	0.773	0.715	0.777
Nasal macrophages	DT (LightGBM feature list)	70	0.731	0.64	0.727
	RF (LightGBM featue list)	1760	0.870	0.825	0.870
Lung macrophages	DT (LightGBM feature list)	100	0.733	0.660	0.733
	RF (LightGBM feature list)	110	0.838	0.795	0.839
Alveolar epithelial cells	DT (mRMR feature list)	1,470	1.000	1.000	1.000
	RF (mRMR feature list)	1,660	0.873	0.838	0.873
Lung endothelial cells	DT (LightGBM feature list)	60	0.753	0.678	0.750
	RF (LightGBM feature list)	170	0.924	0.901	0.924

For blood T cells, Figures 3A, B show the IFS curves of DT and RF on five feature lists. From Figure 3A, DT classifier with top 1,060 features in the mRMR feature list can generate perfect performance with weighted F1 = 1. For RF, the best performance with weighted F1 = 0.971 was obtained using top 1,220 features in the mRMR feature list (Figure 3B). The detailed performance of these two classifiers is provided in Table 2. It is amazing that this DT classifier provided better performance than the RF classifier.

For Nasal B cells, the IFS curves of DT and RF on five feature lists are shown in Figures 4A, B, respectively. When using DT as the classification algorithm, its best performance was obtained by using top 1900 features in the MCFS feature list (Figure 4A). In this case, DT yielded the weighted F1 of 0.610. As for the other classification algorithm, RF, it can be observed from Figure 4B that the top 1,520 features in the MCFS features can support it in producing the best weighted F1 of 0.779. The detailed performance of above DT and RF classifiers is listed in Table 2. Generally, RF classifiers in this cell type on different feature lists were better than DT classifiers.

For Nasal T cells, the IFS curves of DT and RF on five feature lists are provided in Figures 5A, B, respectively. By observing Figure 5A, DT yielded the highest weighted F1 of 0.607 when top 80 features in the LightGBM feature list were adopted. For RF, its highest performance with weighted F1 of 0.773 was accessed when top 1,040 features in the MCFS feature list were used (Figure 5B). Table 2 also shows the detailed performance of above DT and RF classifiers. Evidently, RF classifiers on different lists were superior to DT classifiers according to the IFS results on this cell type.

For Nasal macrophages, the IFS curves of DT and RF on five feature lists are shown in Figures 6A, B. By observing the five IFS curves of DT, as shown in Figure 6A, the highest weighted F1 was 0.731, which was obtained by using top 70 features in the LightGBM feature list. With the same operation, the highest weighted F1 of RF was 0.870 when top 1760 features in the LightGBM feature list were employed. The detailed performance of above DT and RF classifiers is also listed in Table 2. Again, the RF classifiers on different lists provided the better performance than DT classifiers.

For Lung macrophages, IFS curves of DT and RF are illustrated in Figures 7A, B, respectively. With the same arguments, DT and RF yielded the highest performance when top 100 and 110, respectively, features in the LightGBM feature list were used. They yielded the weighted F1 of 0.733 and 0.838, respectively. Detailed performance of such two classifiers is listed in Table 2. RF classifiers on different lists also generated better performance than DT classifiers.

#### 3.2.2 IFS results of tissue cells

For alveolar epithelial cells, the IFS curves of DT and RF on five feature lists are provided in Figures 8A, B, respectively. For DT, it can yield the highest weighted F1 of 1.000 (i.e., the perfect performance) when top 1,470 features in the mRMR feature list were used, which can be observed from Figure 8A. As for RF, its best performance was obtained by using top 1,660 features in the mRMR feature list, which produced the weighted F1 of 0.873 (Figure 8B). The detailed performance of above two classifiers is listed in Table 2. Although above DT classifier was better than above RF classifier, the optimal DT classifiers on other four feature lists were generally weaker than the optimal RF classifiers on the same feature list.

For lung endothelial cells, Figure 9A shows the IFS curves of DT on five feature lists. It can be observed that DT yielded the best performance with weighted F1 of 0.753 when top 60 features in the LightGBM feature list were adopted. As for RF, its IFS curve is provided in Figure 9B, from which we can see that the highest weighted F1 was 0.924. Such performance was obtained by using top 170 features in the LightGBM feature list. The detailed performance of above DT and RF classifiers is listed in Table 2. Clearly, the RF classifier was superior to DT classifier. Furthermore, from Figure 9, DT classifiers were evidently weaker than RF classifiers on the same feature list.

#### 3.2.3 Intersection of different feature lists

According to Figures 2–9, several optimal classifiers employed lots of top features in the corresponding lists. In this case, their efficiencies were not very high. For each of such classifiers, we want to find out another classifier which adopted much less features, whereas its performance was a little lower than the optimal classifier. These classifiers were called feasible classifiers for convenience. The difference on the performance of feasible and optimal classifiers on different feature lists for eight cell types is provided in Table 3 (if exist). It can be observed that the weighted F1 of one feasible classifier was very close to that of the optimal classifier. The proportions were higher than 90%. However, the features used in feasible classifiers were much less than those used in the optimal classifiers. Most proportions were lower than 40%. Such results further indicated that features used in feasible classifiers were most important, which can capture the essential differences on immune responses between different vaccination strategies.

**TABLE 3:** Difference between feasible and optimal classifiers on five feature lists for eight cell types.

Cell Type	Feature list	Classification algorithm	Proportion to the optimal classifier	
			Number of features	Weighted F1
Blood B cells	LASSO feature list	RF	13.33%	98.52%
	LightGBM feature list	RF	25.00%	98.66%
	MCFS feature list	RF	21.62%	98.20%
	mRMR feature list	RF	50.00%	98.06%
	PFI feature list	RF	19.35%	98.53%
Blood T cells	LASSO feature list	RF	31.25%	97.32%
	LightGBM feature list	RF	11.48%	98.17%
	MCFS feature list	RF	6.25%	96.63%
	mRMR feature list^a^	-	-	-
	PFI feature list	RF	42.86%	98.80%
Nasal B cells	LASSO feature list	RF	10.20%	95.12%
	LightGBM feature list	RF	37.50%	97.87%
	MCFS feature list	RF	19.08%	90.12%
	mRMR feature list	RF	26.67%	94.38%
	PFI feature list	RF	43.75%	96.24%
Nasal T cells	LASSO feature list	RF	27.27%	95.29%
	LightGBM feature list^a^	-	-	-
	MCFS feature list	RF	42.31%	97.54%
	mRMR feature list	RF	27.78%	95.94%
	PFI feature list	RF	29.41%	98.20%
Nasal macrophages	LASSO feature list	RF	65.07%	95.94%
	LightGBM feature list	RF	2.84%	99.08%
	MCFS feature list	RF	7.00%	96.52%
	mRMR feature list	RF	26.67%	98.61%
	PFI feature list	RF	5.00%	97.56%
Lung macrophages	LASSO feature list	RF	4.09%	94.77%
	LightGBM feature list	RF	54.55%	99.16%
	MCFS feature list	RF	13.43%	99.14%
	mRMR feature list	RF	26.47%	97.29%
	PFI feature list	RF	14.71%	96.98%
Alveolar epithelial cells	LASSO feature list	RF	6.08%	93.81%
	LightGBM feature list	RF	23.81%	99.15%
	MCFS feature list	RF	36.00%	96.70%
	mRMR feature list^a^	-	-	-
	PFI feature list	RF	61.54%	98.87%
Lung endothelial cells	LASSO feature list	RF	5.82%	90.84%
	LightGBM feature list	RF	23.53%	98.92%
	MCFS feature list	RF	13.11%	95.50%
	mRMR feature list	RF	21.21%	97.38%
	PFI feature list	RF	32.14%	99.12%

^a^ Feasible classifier on the corresponding feature list was not identified.

**FIGURE 10:**
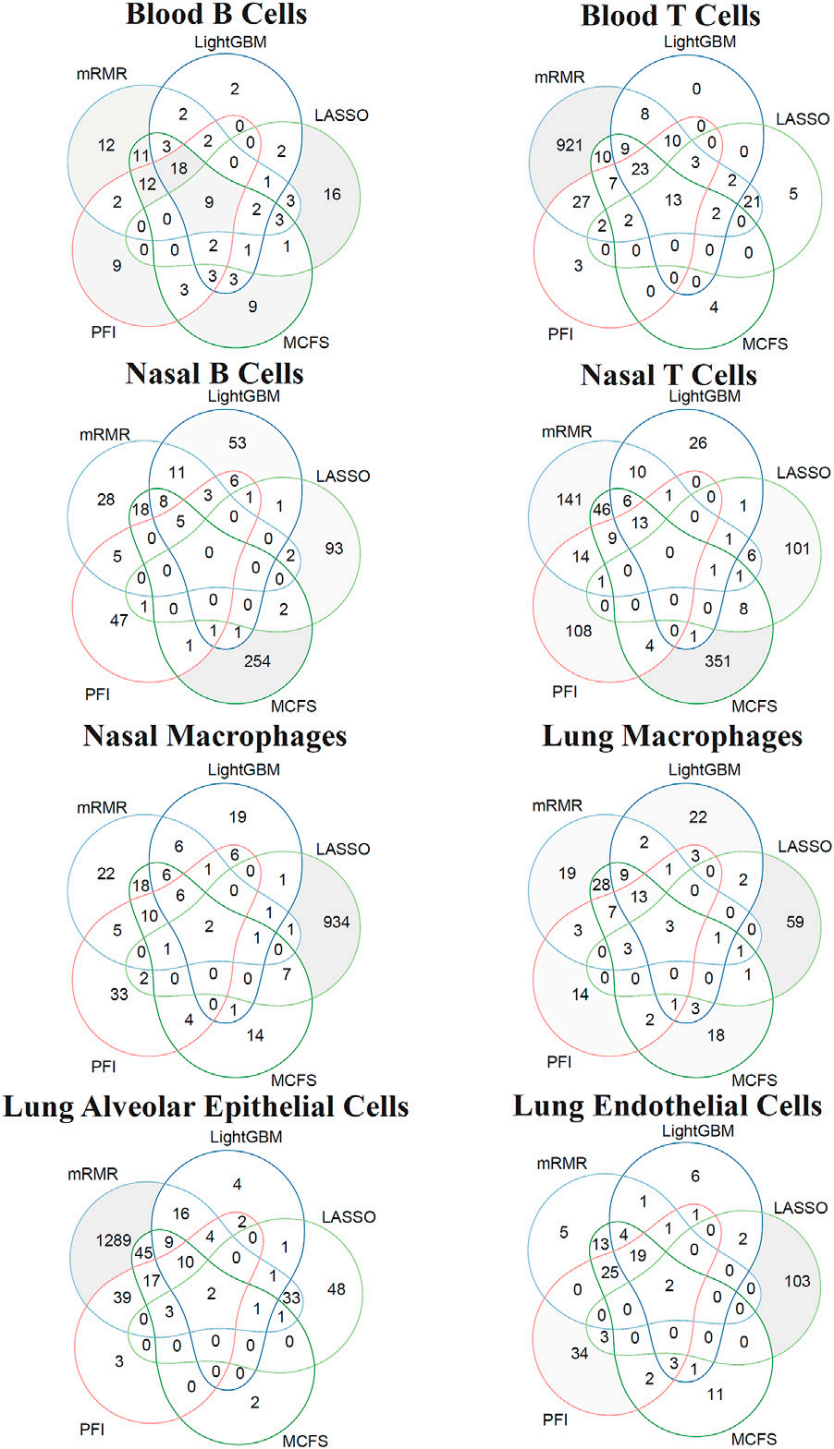
Venn diagram of the features used in feasible classifiers on five feature lists that were generated by LASSO, LightGBM, MCFS, mRMR, and PFI for eight cell types. The overlapping circles indicated genes that were identified to be important by multiple ranking algorithms.

For each cell type, different features were used in the feasible classifiers on different feature lists. Some features may be adopted in multiple feasible classifiers, which can be deemed as more important than others. To show the relationship between five feature subsets used in five feasible classifiers (if feasible classifier was not available, optimal classifier was used), a Venn diagram was plotted for each cell type, as shown in Figure 10. The intersection results for eight cell types are presented in Supplementary Table S3. Some gene features occurred in multiple feature subsets would be analyzed in Section 4.1.

### 3.3 Classification rules

Based on the IFS curves shown in Figures 2–9, the performance of DT classifiers is generally lower than that of RF classifiers. However, as mentioned in the introduction of DT (Section 2.5.2), the interpretability of DT classifiers for prediction can help us analyze their biological significance, which cannot be obtained from RF classifiers. Based on the optimal DT classifiers on different feature lists for each cell type, we extracted the number of optimal features for these DT classifiers. These features were used to represent each sample and a large tree was learned from such representation of all samples. A group of quantitative classification rules can be extracted from such tree. Supplementary Tables S4–11 provide the rule groups yielded by DT on different feature lists for eight cell types. Each rule contained several conditions and one result, describing the expression levels of genes under the corresponding vaccination strategies.

## 4 Discussion

In this study, we integrated multiple machine learning approaches to perform in-depth analysis of single-cell transcriptome data under different COVID-19 vaccine strategies using hamsters as experimental subjects. The effectiveness of various COVID-19 vaccination techniques to provide protection is closely correlated with the gene expression patterns of certain immune and tissue cells. Several optimal classifiers were constructed, which can be used to predict vaccination strategies for two doses of adenovirus vaccine, two doses of attenuated virus vaccine, two doses of mRNA vaccine, one dose of mRNA and one dose of attenuated vaccine. The tissue cells included alveolar epithelial and endothelial cells from the lungs, whereas the immune cells included B cells, T cells, and macrophages from the blood, nasal, and lungs, respectively. Some essential gene features identified by the computational analysis might be crucial and the classification rules can imply the expression levels of key genes in different vaccine strategies after SARS-CoV-2 infection. Thus, the features and rules identified in this study may provide evidence for the immune memory capacity of different vaccination strategies and help advance more effective vaccination methods to combat SAR-CoV-2 infection. Based on the newly released publications, some essential gene features and quantitative rules can be confirmed to play crucial roles in anti-viral responses.

### 4.1 Analysis of top features in SARS-CoV-2-infected hamsters for distinguishing different vaccination strategies

Based on our computational analysis, we identified a set of essential genes differentially expressed in immune cells and lung tissue cells to identify vaccine recipients with different prime-boost vaccination after SARS-CoV-2 infection. Recent studies have demonstrated the mechanism of some genes in the antiviral process. One or two genes were selected for detailed analysis for each cell type, which are listed in Table 4.

**TABLE 4:** Top features identified by the computational analysis in immune and lung cells.

Classification	Cell type	Gene symbol	Description
Immune cells	Blood B cells	RPS23	ribosomal protein S23
		TPT1	tumor protein, translationally-controlled 1
	Nasal B cells	IFIT3	interferon induced protein with tetratricopeptide repeats 3
	Blood T cells	EEF1A1	eukaryotic translation elongation factor 1 alpha 1
		UBA52	ubiquitin A-52 residue ribosomal protein fusion product 1
	Nasal T cells	DDX5	DEAD-box helicase 5
		DEF6	DEF6 guanine nucleotide exchange factor
	Lung macrophages	PFN1	profilin 1
		RPSA	ribosomal protein SA
	Nasal macrophages	ISG15	ISG15 ubiquitin like modifier
Lung cells	Alveolar epithelial cells	IRF9	interferon regulatory factor 9
	Lung endothelial Cells	MX1	MX dynamin like GTPase 1
		MX2	MX dynamin like GTPase 2

#### 4.1.1 Top features in immune cells

In blood B cells, *RPS23* (ENSG00000186468) is a 40S ribosomal protein (Barrado-Gil et al., 2020) that plays a role in ribosome assembly and protein translation, which may be related to antibody production by B cells. Moreover, *RPS23* plays an important role in physiological and pathological processes such as tumorigenesis, immune signaling, and development (Zhou et al., 2015). *RPS23* has also been reported to be a new antimicrobial peptide that can recognize and kill potential pathogens (Ma et al., 2020). Furthermore, the expression level of RPS23 may be related to the immune response induced after vaccination. Two recent studies have found that *RPS23* expression was changed after inactivated vaccination (Pisano et al., 2021), indicating its potential role in immune response. *TPT1* (ENSG00000133112) is involved in the regulation of apoptosis (Bruneel et al., 2005), and it is also related to the regulation of protein synthesis in immune cells (Arowolo et al., 2021). Moreover, *TPT1* is involved in the viral response (Leong and Chow, 2006). Based on recent publications, *TPT1* plays an important role in the development of COVID-19 (Hasankhani et al., 2021), and it can be used to predict COVID-19 (Akbulut et al., 2022). Therefore, *TPT1* may be involved in the antiviral response induced by SARS-CoV-2 infection, thereby promoting the exploration of the immune memory capacity induced by different vaccines.

In nasal B cells, *IFIT3* (ENSG00000119917) belongs to the interferon-stimulated gene (ISG) family, and it is involved in immune processes, including innate immunity, inflammatory response, and antiviral immunity (de Veer et al., 1998; Fleith et al., 2018). In addition, *IFIT3* is differentially expressed in B cells and monocytes in patients with autoimmune diseases (Fang et al., 2021), indicating that the *IFIT3* gene may be involved in B cell-mediated humoral immunity. With regard to the relationship between *IFIT3* and viral infection, *IFIT3* was found to be differentially expressed in response to infection with RNA viruses (Zhou et al., 2013; Feng et al., 2018) and was considered to have predictive potential for COVID-19 because the expression level can be affected by SARS-CoV-2 infection (Shaath et al., 2020; Gao et al., 2021).

In blood T cells, *EEF1A1* (ENSG00000156508) encodes the same type of alpha subunit of a complex, namely, elongation factor-1, which is responsible for aminoacyl tRNAase delivery to the ribosome; promotes cell growth and proliferation; and inhibits apoptosis (Mills and Gago, 2021). Huang et al. found that the expression of *EEF1A1* was positively correlated with the number of initial CD4^+^ T cells (Huang and Zhou, 2022), indicating that *EEF1A1* may be associated with cellular immunity. In addition, *EEF1A1* could inhibit viral growth (Zhang et al., 2015), and it is associated with inflammatory responses (Maruyama et al., 2007). The *EEF1A1* protein has been reported to play a key role in several viral infections by interacting with viral proteins (Sikora et al., 2009; Zhang et al., 2015). Based on a recent study, SARS-CoV-2 infection affects *EEF1A1* expression, and it may be associated with the suppression of viral RNA replication. Ubiquitin A-52 residue ribosomal protein fusion product 1, *UBA52* (ENSG00000221983), is a ubiquitin-encoding gene encoding ubiquitin fusion proteins (Kobayashi et al., 2016). *UBA52* participates in H5N1 viral replication (Wang et al., 2018), which is linked to viral infection. *UBA52* deficiency may cause cell cycle arrest and inhibit protein synthesis (Mao et al., 2018), revealing its potential role in T cells performing antiviral functions. In addition, *UBA52*, as a ubiquitin-encoding gene, might be associated with antigen processing and MHC II antigen presentation, which is consistent with the role of *UBA52* in the proteasomal degradation of CD4^+^ T cells after SARS-CoV-2 infection identified by Tiwari et al. (Tiwari et al., 2022).

In nasal T cells, *DDX5* (ENSG00000108654), also known as p68, is a typical member of the dead box ATP-dependent RNA unwinding enzyme family (Lane and Hoeffler, 1980). *DDX5* gene encodes a protein that plays an important role in RNA metabolism (Zonta et al., 2013; Dardenne et al., 2014). A recent study has focused on the function of *DDX5* in regulating cellular life cycles, cancer and development, and spermatogenesis (Hashemi et al., 2019; Legrand et al., 2019; Hu et al., 2022). Notably, *DDX5* has been associated with multiple viral infections. For example, *DDX5* could inhibit RNA transcription of hepatitis B virus (Zhang et al., 2016) and enhance RNA transcription of hepatitis C virus (Goh et al., 2004), and *DDX5* may promote SARS-CoV replication (Chen J. Y. et al., 2009). In addition, a recent study has found that *DDX5* is involved in the regulation of SARS-CoV-2 replication (Ariumi, 2022), thereby identifying the ability of the immune memory of COVID-19 vaccine. *DEF6* (ENSG00000023892), also known as IRF4-binding protein or SWAP-70-like bridging protein (SLAT) of T cells, is a specific guanine nucleotide exchange factor for Rho GTPase Cdc42 and Rac1 (Deng et al., 2020). *DEF6* is expressed in myeloid cells, and it controls innate immunity (Chen Q. et al., 2009). Thus, it is strongly related to immunity. Moreover, mutations or deletions of *DEF6* can lead to immune dysregulation diseases (Fournier et al., 2021). *DEF6*, as a feature gene, is highly expressed in T cells, and it plays an important role in T cell proliferation, Th1/Th2 lineage differentiation, and function. It is also involved in T cell receptor signaling regulation (Izawa et al., 2017; Deng et al., 2020). Some researchers have also found that *DEF6* deficiency adversely affects the function of memory T cells (Rossi et al., 2011).

In lung macrophages, *PFN1* (ENSG00000108518) is a key actin regulatory protein that is involved in the regulation of actin filament assembly (Mouneimne et al., 2012), which may be related to the migration of macrophages to the site of infection. *PFN1* may also be crucial for viral transcriptional activation and airway hyperresponsiveness (Leng et al., 2021). As *PFN1* expression is altered by SARS-CoV-2 infection (Shen et al., 2020), it can be identified as a biomarker to detect COVID-19. *RPSA* (ENSG00000168028) is an important component of the small ribosomal subunit with a wide range of physiological functions, including RNA processing, cell migration, and angiogenesis (Bernard et al., 2009; O'Donohue et al., 2010; Rea et al., 2012). *RPSA* also plays a role in regulating the mitogen-activated protein kinase (MAPK) signaling pathway (Givant-Horwitz et al., 2004), and many viral infections have been associated with deviations from well-balanced control of the MAPK signaling cascade, such as Ebola virus (Strong et al., 2008) and influenza A virus (Mizumura et al., 2003). *RPSA* has been found to be expressed in a variety of immune cells, including neutrophils, monocytes, and T cells (Sun et al., 2020), to participate in the immune process. In macrophages, *RPSA* expression levels were altered after infection with *Mycoplasma pleuropneumoniae* and porcine circovirus type 2 (Liu M. et al., 2021) or after BCG vaccination (Liu et al., 2022).

In nasal macrophages, an abundantly induced ISG, *ISG15* (ENSG00000187608), is crucial for viral infection (Morales and Lenschow, 2013). In the beginning of the innate response to viral infection, *ISG15* has been shown to be substantially increased as an effector and signaling molecule (Freitas et al., 2020). In addition, *ISG15* can prevent viral replication by interfering with the exocytosis and endogenous translation machinery that viruses rely on to grow (Okumura et al., 2007). Following SARS-CoV-2 infection, a study found that the secretion of *ISG15* exacerbated the inflammatory response (Cao, 2021), indicating the immunological role of *ISG15* in COVID-19. In macrophages, the expression of *ISG15* can promote macrophage polarization toward a pro-inflammatory and antiviral M1 phenotype to produce more antiviral factors (Freitas et al., 2020). Furthermore, macrophages can display increased autophagy and mitophagy of infected cells under *ISG15* stimulation (Swaim et al., 2017).

#### 4.1.2 Top features in lung tissue cells

In lung alveolar epithelial cells, *IRF9* (ENSG00000213928) is a key component of the type I and type III interferon signaling pathways, which controls the antiviral response of cells to type I and type III interferons (Stark and Darnell, 2012; Lazear et al., 2019). The antiviral ability of *IRF9* against common viruses such as respiratory viruses has been well demonstrated (Hernandez et al., 2018; Bravo García-Morato et al., 2019). A recent study revealed that the high expression level of *IRF9* in SARS-CoV-2-infected cells controls the ISGF-3-dependent response to type I and type III interferons, thereby accelerating the initiation of the immune response (Ahmed, 2020). Therefore, the expression level of the *IRF9* gene is related to the degree of SARS-CoV-2 infection of alveolar epithelial cells.

In lung endothelial cells, *MX1* (ENSG00000157601) and *MX2* (ENSG00000183486) encode two different guanosine triphosphate (GTP)-metabolizing proteins that differ remarkably in viral specificity and mechanism of action. *MX1* has a wide antiviral activity against RNA and DNA viruses, whereas *MX2* is only effective against certain viruses, such as HIV (Jung et al., 2019). *MX1* is involved in the antiviral innate response, and it regulates neutrophil activity and brings neutrophils into the tissues for immune functions (Henarejos-Castillo et al., 2020). *MX1* can be induced by SARS-CoV-2 infection (Senapati et al., 2020; Halfmann et al., 2022). Based on a study conducted in 2020, SARS-CoV-2 can induce strong expression of *MX1* in the lungs of infected hamsters (Halfmann et al., 2022). Thus, the expression of *MX1* and *MX2* could be used to determine the degree of lung infection.

### 4.2. Analysis of Classification Rules in SARS-CoV-2-infected Hamsters for Distinguishing Different Vaccination Strategies

Besides essential genes, quantitative rules were another main output of the computational analysis, which are provided in Supplementary Tables S4–11. Each rule contained several gene features and thresholds. It is quite difficult to confirm the underlying expression patterns of each rule. Here, we extracted some important conditions for detailed analysis. For each cell type, we focused on one important gene such that different results (class labels) can be outputted with different thresholds and tendencies. The conditions for each cell type are listed in Table 5.

**TABLE 5:** Representative conditions for different cell types.

Cell Type	Rules	Parameters	Predicted class
Blood B cells	Condition 0	ENSG0000019609(PAX5) ≤ 2.1383	Non-vaccinated
	Condition 1	ENSG0000019609(PAX5) > 0.3466	2*Attenuated
	Condition 2	ENSG0000019609(PAX5) > 0.8959	mRNA/Attenuated
Nasal B cells	Condition 3	ENSG00000119917(IFIT3) > 0.3466	Non-vaccinated
	Condition 4	ENSG00000119917(IFIT3) > 0.8959	mRNA/Attenuated
Blood T cells	Condition 5	ENSG00000221983(UBA52) ≤ 2.4414	Non-vaccinated
	Condition 6	ENSG00000221983(UBA52) >2.4414	2*Adenovirus
	Condition 7	ENSG00000221983(UBA52) > 2.1910	2*Attenuated
	Condition 8	ENSG00000221983(UBA52) > 2.7403	mRNA/Attenuated
Nasal T cells	Condition 9	ENSG00000233927(RPS28) ≤ 4.0687	Non-vaccinated
	Condition 10	ENSG00000233927(RPS28) > 4.0687	2*Adenovirus
	Condition 11	ENSG00000233927(RPS28) > 4.0687	2*Attenuated
Lung macrophages	Condition 12	ENSG00000163563(MNDA) > 3.5972	Non-vaccinated
	Condition 13	ENSG00000163563(MNDA) ≤ 3.2385	2*Adenovirus
	Condition 14	ENSG00000163563(MNDA) ≤ 3.5972	2*mRNA
	Condition 15	ENSG00000163563(MNDA) ≤ 2.8029	mRNA/Attenuated
Nasal macrophages	Condition 16	ENSG00000160932(LY6E) > 2.5249	Non-vaccinated
	Condition 17	ENSG00000160932(LY6E) ≤ 2.5249	2*Adenovirus
	Condition 18	ENSG00000160932(LY6E) ≤ 2.5249	2*Attenuated
	Condition 19	ENSG00000160932(LY6E) ≤ 2.5249	2*mRNA
	Condition 20	ENSG00000160932(LY6E) ≤ 2.5249	mRNA/Attenuated
Lung alveolar epitheial cells	Condition 21	ENSG00000187608(ISG15) > 0.8959	Non-vaccinated
	Condition 22	ENSG00000187608(ISG15) ≤ 0.8959	2*Adenovirus
	Condition 23	ENSG00000187608(ISG15) ≤ 0.8959	2*Attenuated
Lung endothelial cells	Condition 24	ENSG00000204264(PSMB8) > 0.3466	Non-vaccinated
	Condition 25	ENSG00000204264(PSMB8) ≤ 1.9945	2*Attenuated

#### 4.2.1 Classification rules in immune cells

In blood B cells, *PAX5* (ENSG00000196092) is upregulated in samples with two doses of attenuated vaccination and mRNA/attenuated vaccination but downregulated in unvaccinated samples. *PAX5* is a crucial gene, which is known as a key factor for B cell proliferation and differentiation (Mullighan et al., 2007). Harris et al. found that *PAX5* binds to Fbxo7 transcription in pre-B cells (Harris et al., 2021). *FBX O 7* is known for its important role in lymphocyte development and differentiation (Ballesteros Reviriego et al., 2019). Thus, *PAX5* might be involved in the positive regulation of B cell proliferation and differentiation. The expression of *PAX5* is essential for memory B cell development after antigen encounter (Johnson et al., 2005; Nutt and Tarlinton, 2011). In addition, *PAX5* expression declines as plasma cells differentiate (Urbánek et al., 1994; Cobaleda et al., 2007), which may partially reflect immunological memory activation. Based on our classification rules, the expression of *PAX5* in B cells may indicate that specific vaccine combinations induce better B cell memory.

In nasal B cells, *IFIT3* (ENSG00000119917) was identified by our computational method, which was shown to be upregulated in unvaccinated and heterologous vaccinated samples. However, the upregulation of *IFIT3* expression was remarkable in mRNA/attenuated vaccination samples. *IFIT3* was found to be involved in viral responses (Metz et al., 2013). *IFIT3* is an IFN-inducible protein whose expression is increased by viral infection and IFN treatment (Pidugu et al., 2019). Although no direct evidence is found for the role of *IFIT3* expression in B cells, altered *IFIT3* expression induced by SARS-CoV-2 infection has been widely demonstrated. *IFIT3* could be related to immune response to SARS-CoV-2 infection based on the findings of several studies, demonstrating that *IFIT3* is strongly expressed in the pulmonary inflammatory cells of patients with COVID-19 (Shaath et al., 2020; Vishnubalaji et al., 2020). Moreover, *IFIT3* was found to play an important role in limiting the replication of RNA viruses, including SARS-CoV-2 (Metz et al., 2013; Pfaender et al., 2020; Martin-Sancho et al., 2021). Collectively, the expression level of *IFIT3* may indicate the immune response to viral infection in B cells, which can be used to compare the immunological memory induced by various vaccination strategies.

In blood T cells, *UBA52* (ENSG00000221983) was identified as a rule gene. *UBA52* expression in T cells was shown to be upregulated in recipients with two doses of adenovirus vaccination, two doses of attenuated vaccination, and mRNA/attenuated vaccination. As previously discussed, *UBA52* was considered as a signature gene in blood T cells. As a ubiquitin-encoding gene (Kobayashi et al., 2016), *UBA52* was found to be closely associated with proteasomal degradation in CD4^+^ T cells(V'Kovski et al., 2019). Picciotto et al. indicated that *UBA52* is rapidly upregulated after T-cell activation (de Picciotto et al., 2022), and it may be involved in effector T-cell activation. In addition, *UBA52* was found to be highly expressed in patients with COVID-19 (Jiang et al., 2022), which may be related to COVID-19 pathogenesis. Thus, the differential expression of *UBA52* in blood T cells helps to distinguish different prime-boost vaccination strategies.

In nasal T cells, ribosomal gene *RPS28* (ENSG00000233927) was identified, whose basic function is to participate in protein synthesis, folding, and assembly (Kim et al., 2019). Based on our rules, *RPS28* was upregulated in samples with two doses of adenovirus vaccine and two doses of attenuated vaccine. *RPS28* has been reported to control the generation of MHC class I peptides by regulating non-canonical translation (Wei et al., 2019), leading to differential antigen presentation in cells. A study on melanoma found that mutations in ribosomal proteins resulting in the deletion of *RPS28* caused greater killing of melanoma cells by CD8^+^ T cells (Dersh et al., 2021), indicating the association of *RPS28* with CD8^+^ T cells. Therefore, the expression of *RPS28* in nasal T cells may help to distinguish different vaccine combinations and predict immune memory activation.

In lung macrophages, *MNDA* (ENSG00000163563) was identified, whose function is thought to be related to immune cells (Metcalf et al., 2014). *MNDA* was downregulated in samples receiving COVID-19 vaccines, with the greatest downregulation in mRNA/attenuated vaccine recipients. *MNDA* is an interferon-inducible gene, whose protein contains a pyridine structural domain that plays a role in programmed cell death and inflammation-related signaling (Bottardi et al., 2020). *MNDA* was strongly expressed in activated macrophages linked to inflammation but not in normal tissue cells (Miranda et al., 1999), indicating the relationship between *MNDA* expression and tissue inflammation. In monocytes, *MNDA* was found to be remarkably upregulated after IFNα exposure (Briggs et al., 1994), and it could be a major regulator of monocyte and granulocyte lineage (Milot et al., 2012). Thus, the downregulation of the immune-related gene *MNDA* in lung macrophages may be due to the good protective capacity of the vaccine to keep the lungs free from viral infection.

In nasal macrophages, *LY6E* (ENSG00000160932) was identified as a rule gene, whose expression was shown to be downregulated in all vaccination strategies except for controls. *LY6E* encodes an interferon-inducible protein, which has been shown to regulate viral infection in a cell type-dependent manner (Godfrey et al., 1992). *LY6E* is involved in the regulation of infection by a variety of viruses, and it was found to promote HIV-1 (Yu et al., 2017), yellow fever virus (Schoggins et al., 2011), and influenza A virus (Mar et al., 2018) infection. Therefore, the reduced expression of the *LY6E* gene in nasal macrophages of samples with COVID-19 vaccination may be due to the fact that COVID-19 vaccination helped to avoid SARS-CoV-2 attack on the nasal cavity.

#### 4.2.2 Classification rules in lung tissue cells

In lung alveolar epithelial cells, *ISG15* (ENSG00000187608) was identified as a rule gene in lung alveolar epithelial cells, which is an IFNα-stimulated gene that plays an important role in the antiviral response (Swaim et al., 2020). *ISG15* was downregulated in samples with two doses of adenovirus or attenuated vaccination and upregulated in controls, reflecting the protective ability of COVID-19 vaccination on target cells. It is hypothesized that *ISG15* can prevent viral assembly by tagging newly translated viral proteins (Shin et al., 2020). *ISG15* has also been found to drive antiviral immune functions by modifying viral proteins, inhibiting viral replication, and regulating host signaling pathways associated with viral infection (Perng and Lenschow, 2018). In addition, *ISG15* expression exacerbates the inflammatory response of COVID-19 (Cao, 2021), partially indicating the tissue damage caused by SARS-CoV-2 infection. Thus, the expression of *ISG15* on alveolar epithelial cells may reflect virus-induced damage, helping to compare the protective capacity of COVID-19 vaccines.

In lung endothelial cells, the *PSMB8* (ENSG00000204264) gene was found to be upregulated in controls and downregulated in samples receiving two doses of attenuated COVID-19 vaccines based on our rule. *PSMB8* encodes the proteasome 20S subunit Beta 8, and it is involved in the positive regulation of apoptosis (Yang et al., 2009; Jean-Baptiste et al., 2017). More et al. found that *PSPM8* is involved in mediating viral infection and synthesis in target cells, indicating the potential role of *PSPM8* in viral infection (More et al., 2019). In addition, *PSMB8* is involved in regulating cytokine secretion during viral infection (Servaas et al., 2021). Furthermore, in patients with mild COVID-19, the high expression of *PSMB8* could promote M1 macrophage polarization (Desterke et al., 2021). The extensive involvement of *PSPM8* in viral infection may help us to identify lung damage caused by SARS-CoV-2 infection.

## 5 Conclusion

In investigating the differences in immune changes induced by SARS-CoV-2 infection under different vaccination strategies, this study designed a machine learning based framework to analyze expression profile datasets from lung tissue cells (endothelial cells and alveolar epithelial cells) and immune cells from different sites (B cells, T cells, and macrophages). Five feature ranking methods and two classification algorithms were used to obtain key genes and easily understand quantitative classification rules associated with COVID-19 vaccination and SARS-CoV-2 infection. These results revealed the pathways of action of different vaccination regimens in COVID-19, which could lead to the development of safe and long-lasting vaccination regimens.

## Data Availability

Publicly available datasets were analyzed in this study. This data can be found here: https://www.ncbi.nlm.nih.gov/geo/query/acc.cgi?acc=GSE200596.
